# 1403. Facilitators and barriers to latent tuberculosis infection diagnosis and care in Massachusetts: a convergent mixed methods study of clinicians

**DOI:** 10.1093/ofid/ofac492.1232

**Published:** 2022-12-15

**Authors:** Jeffrey Campbell, Yamini Adusumelli, Mary Tabatneck, Susan N Sherman, Gabriella S Lamb, Vishakha Sabharwal, Don Goldmann, Alexandra Epee-Bounya, Jessica Haberer, Thomas J Sandora

**Affiliations:** Boston University School of Medicine, Boston, Massachusetts; Boston University School of Medicine, Boston, Massachusetts; Boston Children’s Hospital, Boston, Massachusetts; SNS Research, Cincinnati, Ohio; Boston Children's Hospital, Boston, Massachusetts; Boston University, Boston, Massachusetts; Harvard Medical School, lexington, Massachusetts; Boston childrens' Hospital, Boston, Massachusetts; Massachusetts General Hospital, Boston, Massachusetts; Boston Children's Hospital, Boston, Massachusetts

## Abstract

**Background:**

Understanding barriers and facilitators to latent tuberculosis infection (LTBI) diagnosis and care is needed to successfully treat children/adolescents with LTBI in the US. We explored physicians’ perspectives on pediatric LTBI diagnosis and care, and strategies to improve care.

**Methods:**

We conducted a convergent mixed methods study with physicians in Massachusetts. Participants were purposefully sampled from primary care clinics (n=10), clinics seeing immunocompromised patients (n=2), and TB clinics (n=2). Physicians participated in individual qualitative semi-structured interviews exploring experience and comfort with LTBI care, and perceived barriers and facilitators to care. We used applied thematic analysis to analyze transcripts. Participants completed surveys to assess comfort with LTBI care and volume of LTBI patients in their care.

**Results:**

Of the 25 physicians invited, 14 participated. Most participants reported “medium” or “high” comfort with current LTBI guidelines; volume of LTBI care varied by physician type (**Table 1**). Analysis revealed perceived barriers (**Figure 1**) at four steps of care: 1) identification of risk and testing for LTBI (e.g., family/patient risk perception, physician knowledge gaps), 2) completion of referral after a positive test (e.g., communication barriers), 3) treatment acceptance and initiation (e.g., lack of social support), and 4) treatment adherence and completion (e.g., adolescents’ emerging autonomy). Facilitators such as protocolized screening, counseling strategies, free medication, and telehealth (**Figure 2**) overcame some barriers. Important emergent themes included: 1) COVID-19 has induced rapid positive and negative changes in LTBI care in primary care clinics; 2) immigrant adolescents are uniquely at risk for disengagement due to lack of social support; and 3) physicians and clinics are ill-equipped to provide TB care for patients’ close contacts, despite knowledge of need for care.

**Table 1.**
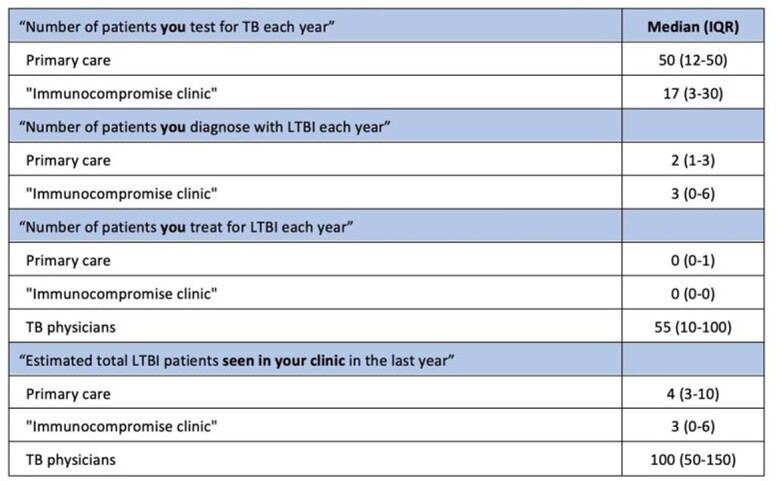
Volume of patients by clinician type (N=13 respondents)

**Figure 1.**
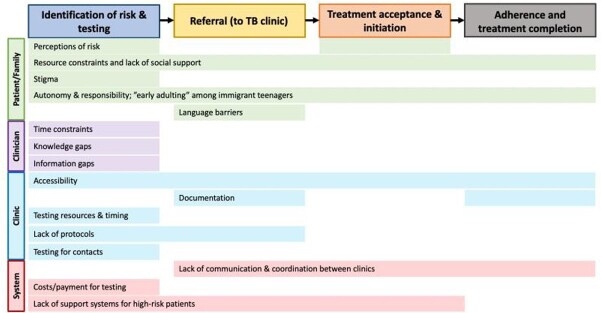
Barriers to LTBI diagnosis and care.

**Figure 2.**
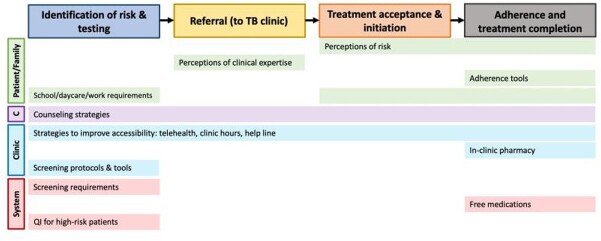
Facilitators of LTBI care.

**Conclusion:**

Lack of perceived risk, family and clinic resource constraints, and accessibility challenges hindered LTBI care; protocolized screening, telehealth, and free medications were among the facilitators that overcame some but not all barriers. These results will inform improvement of LTBI care within and between clinics.

**Disclosures:**

**Jessica Haberer, MD, MS**, Merck: Advisor/Consultant|Natera: Stocks/Bonds.

